# Bronchoesophageal Fistula After Systematic Mediastinal Lymph Node Dissection With Pulmonary Lobectomy

**DOI:** 10.1016/j.atssr.2022.09.013

**Published:** 2022-09-29

**Authors:** Terumoto Koike, Yuta Hosoda, Masaya Nakamura, Yuki Shimizu, Tatsuya Goto, Ken-Ichi Mizuno, Masanori Tsuchida

**Affiliations:** 1 Division of Thoracic and Cardiovascular Surgery, Niigata University Graduate School of Medical and Dental Sciences, Niigata, Japan; 2 Division of Gastroenterology and Hepatology, Niigata University Graduate School of Medical and Dental Sciences, Niigata, Japan

## Abstract

We present a case of a bronchoesophageal fistula after a lobectomy with systematic mediastinal lymphadenectomy for lung adenocarcinoma. A 70-year-old woman was readmitted with postprandial cough, fever, and dysphagia on postoperative day 13. Computed tomography revealed a bronchoesophageal fistula between the left main bronchus and esophagus. Esophagogastroscopy revealed a 3-mm fistula 30 cm from the incisors. Two rounds of endoscopic closure with metal clips were performed. The patient has survived for 24 months with no evidence of recurrent fistula. Although postoperative ischemic bronchitis after mediastinal lymphadenectomy may cause bronchoesophageal fistulas, they can be treated with endoscopic closure.

Postoperative ischemic bronchitis (POIB) after systematic extensive mediastinal lymph node dissection (MLND) with pulmonary resection is a frequent and potentially life-threatening complication.^1^ A bronchopleural fistula is a relatively common complication that is closely related to POIB after pulmonary resection with MLND,^2^ and patients with bronchopleural fistulas usually experience pneumothorax and empyema. We present a case of a left bronchoesophageal fistula with subsequent aspiration pneumonia occurring after a right lower lobectomy with systematic MLND for lung adenocarcinoma. The patient was successfully treated with endoscopic clipping of the esophagus.

This report was approved by the institutional review board of Niigata University Hospital on June 20, 2022 (No. 2022-0016).

A 70-year-old woman was initially admitted to our institution for elective operation for a 2.3-cm clinical stage IA lung adenocarcinoma. She had no history of smoking. Right lower lobectomy with systematic MLND of the superior, subcarinal, and inferior mediastinum was performed (Figure 1A). During MLND, an advanced bipolar energy device (ENSEAL; Ethicon Endo-Surgery) and a modern monopolar soft-coagulation electrosurgical unit (VIO 300D; Elektromedizin) were used for dissection and hemostasis around the trachea and bronchus. Postoperative pathologic examinations revealed multiple node metastases in the hilum and superior and subcarinal mediastinum. The postoperative course was uneventful, and she was discharged on postoperative day 7.

**Figure 1:**
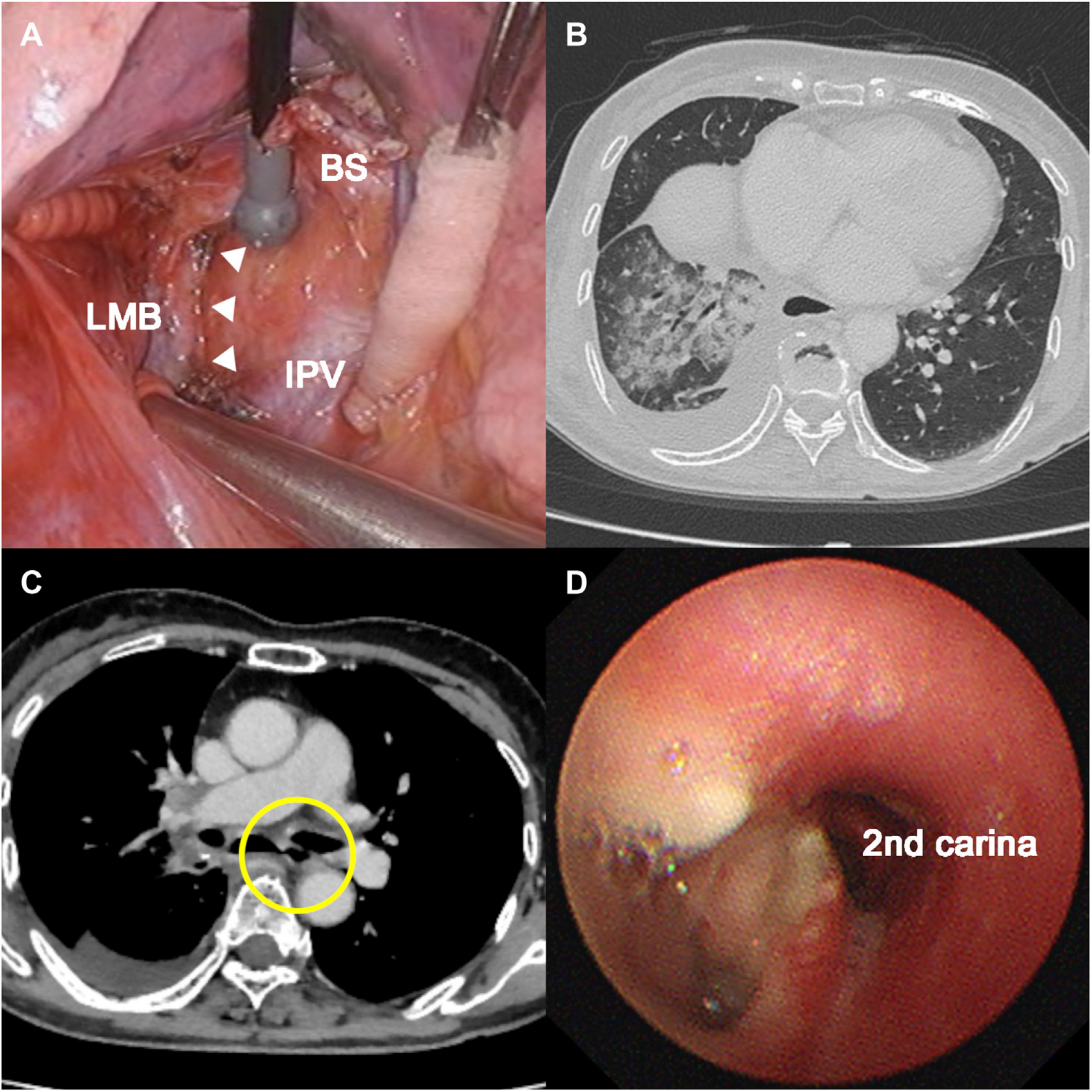
(A) Extensive systematic lymph node dissection of the mediastinum was performed (The arrowheads indicate the left main bronchus). Computed tomography of the thorax revealed (B) extensive infiltrative shadows centered around the right middle lobe without pneumothorax and (C) bronchoesophageal fistula between the left main bronchus and esophagus (circle). (D) Bronchoscopy revealed a small hole at the membranous portion of the left main bronchus. (BS, bronchial stump; IPV, inferior pulmonary vein; LMB, left main bronchus.)

On postoperative day 13, she was readmitted to the hospital with a postprandial cough, fever, and dysphagia. Chest radiography revealed consolidation in the lower right zone of the lungs. Computed tomography of the thorax revealed extensive infiltrative shadows centered around the right middle lobe without pneumothorax (Figure 1B) and a bronchoesophageal fistula between the left main bronchus and the esophagus (Figure 1C). Bronchoscopy revealed a small hole in the membranous portion of the left main bronchus (Figure 1D), and esophagogastroscopy showed a 3-mm fistula 30 cm from the incisors (Figure 2A).

**Figure 2:**
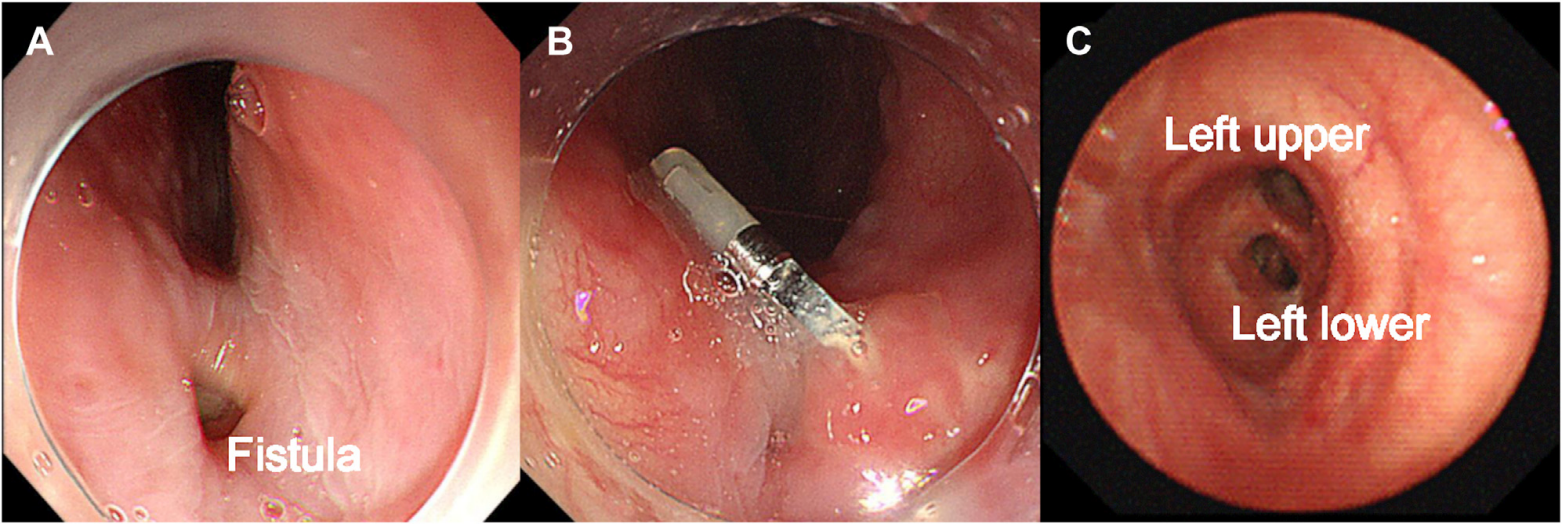
(A) Esophagogastroscopy showed a 3-mm fistula 30 cm from the incisors. (B) After repeated endoscopic clipping, the fistula was closed. (C) Bronchoscopy showed a healed membranous portion of the left main bronchus.

Aspiration pneumonia was diagnosed, the patient was treated with antibiotics, and feeding was initiated through a nasogastric tube. Esophagogastroscopy was performed by a gastroenterologist, and endoscopic fistula closure with metal clips (EZ Clip; Olympus Corporation) was performed. Two weeks after the initial endoscopic treatment, a second esophagogastroscopy showed that the clips had reduced the fistula. Additional endoscopic clipping was performed. On day 23 after the initial endoscopic closure, a third esophagogastroscopy showed that the fistula had closed without any complications, such as stenosis or newly formed fistulas (Figure 2B).

The patient resumed oral eating on day 28 after the initial endoscopic closure. She was discharged from the hospital on day 36 without any signs of recurrence of aspiration pneumonia.

Four months after endoscopic treatment, bronchoscopy revealed a healed membranous portion of the left main bronchus without stenosis (Figure 2C). The patient has survived for 24 months with no evidence of recurrence of the bronchoesophageal fistula or aspiration pneumonia, although she has undergone systematic chemotherapy for multiple lymph node and bone metastases in the thoracic vertebrae.

## Comment

A bronchopleural fistula due to POIB is a complication of systematic extensive MLND with pulmonary resection for lung cancer.^1^ However, occurrence of a bronchoesophageal fistula after pulmonary lobectomy with systematic MLND is rare and has never been reported before.

Extensive MLND enables complete removal of all mediastinal nodes and surrounding fatty tissue and may compromise bronchial microvascularization.^3^ Benhamed and coworkers^1^ reported on POIB after systematic MLND; using fiberoptic bronchoscopy, they found POIB in 34 of 1058 patients (3.21%), including 27 with true ischemia (80%) and 7 with necrosis (20%). POIB was asymptomatic in 27 patients (80%) and was localized to bronchial stumps in most patients, even on the homolateral bronchial tree, and extended toward the contralateral bronchial tree. In our case, extensive MLND, including both superior and inferior mediastinal lymph nodes, was performed. Subcarinal MLND has been suggested as a major factor for POIB.^2^ Thus, we believe that in our case, POIB on the membranous portion of the left main bronchus due to extensive MLND resulted in a bronchoesophageal fistula without a bronchopleural fistula. Moreover, we used an advanced bipolar energy device and modern monopolar soft-coagulation electrosurgical unit for dissection and hemostasis around the trachea and bronchus. The use of such novel electronic devices might affect the development of POIB by bronchial tissue damage, with intense acute inflammation extending deep into the bronchial structure.^4^

Previous studies have reported surgical management for acquired nonmalignant fistulization between the airway and esophagus.^5^ However, surgical repair is challenging and demanding. It requires soft tissue interposition to separate the esophagus and airways, sometimes requires multistage surgery for a recurrent fistula or postoperative complications, and shows relatively high morbidity and probability of postoperative death. Fortunately, in our case, the bronchoesophageal fistula was successfully treated without surgical intervention by repeated esophagogastric endoscopic closure with metal clips. Endoscopic treatment of acquired nonmalignant trachea/bronchoesophageal fistulas, esophageal stenting, airway stenting, and esophagogastric endoscopic clip replacement have been reported^6^; however, the success rate was low (45.5%). In the report, endoscopic closure had a better success rate in treating bronchoesophageal fistula than in treating tracheoesophageal fistula. In addition, the time for fistula healing tended to be longer in cases with previous radiation therapy. Most POIB can be improved and healed without treatment within several months,^2^ as distinct from after radiation therapy; thus, this may explain the success of the endoscopic closure for bronchoesophageal fistula, not tracheoesophageal fistula, in our case.

We report a case of a left bronchoesophageal fistula due to POIB after systematic extensive MLND with right lower lobectomy. Although extensive MLND using novel electronic devices is a potential cause of POIB with bronchoesophageal fistula, it can be treated by endoscopic closure without surgical intervention. However, we still consider novel electronic devices useful for quick and efficient dissection with reduced bleeding and lymphorrhea resulting from the sealing of small vessels. Direct contact between the tip of electronic devices and the bronchial/tracheal walls should be avoided to reduce the risk of POIB.
